# Necessary, but Not Sufficient. The Benefit Concept in the Project Evaluation of Animal Research in the Context of Directive 2010/63/EU

**DOI:** 10.3390/ani8030034

**Published:** 2018-02-28

**Authors:** Matthias Eggel, Herwig Grimm

**Affiliations:** 1 Messerli Research Institute, University of Veterinary Medicine Vienna, Veterinärplatz 1, 1210 Vienna, Austria; herwig.grimm@vetmeduni.ac.at; 2 Institute for Biomedical Ethics and History of Medicine, University of Zurich, Winterthurerstrasse 30, 8006 Zurich, Switzerland

**Keywords:** animal ethics, harm-benefit analysis, animal research ethics, benefit concept

## Abstract

**Simple Summary:**

According to Directive 2010/63/EU, project proposals involving experiments on animals have to be approved in a harm-benefit-analysis (HBA) that weighs the potential benefits of the experiment against the harm inflicted on animals. Only if the benefit outweighs the harm, will the project be approved. However, it is unclear what counts as a valid benefit. In this paper, we analyze the underlying premises of the HBA and its consequences for the project evaluation process. We come to the conclusion that knowledge, as such, is considered a low benefit and that only knowledge applied to benefit society, e.g., new cancer treatment or potent vaccine, etc., is considered to be a high benefit. However, we demonstrate that benefit of this kind cannot be assessed prospectively for research proposals due to the inherent uncertainties of research and the difficulty of determining extra-scientific factors that are crucial for the generation of societal benefit. As a consequence, we advocate a reevaluation of current project evaluation and propose to develop an alternative model for project evaluation.

**Abstract:**

Directive 2010/63/EU (henceforth “Directive”) on the protection of animals used for scientific purposes mandates that every project proposal in EU member states involving procedures on living non-human vertebrates and cephalopods has to be approved in an review process, including a harm-benefit-analysis (HBA), to assess “whether the harm to the animals in terms of suffering, pain and distress is justified by the expected outcome taking into account ethical consideration and may ultimately benefit human beings, animals or the environment”. Despite the justifying relevance of “outcome” and “benefit”, it remains unclear how to understand these concepts. However, national authorities and applicants require a clear understanding of this to carry out a HBA. To analyze the underlying premises of the HBA and its consequences for the evaluation process, we introduce a heuristic to analyze the relation between “outcome”, “benefit” and “prospective benefit assessment”. We then apply the heuristic to all seven legitimate purposes for animal research stated in the Directive, namely basic research, translational or applied research, product safety, education and training, protection of the environment, preservation of species and forensic inquiries. As we show, regardless of which purpose is aimed for, applicants are hard-pressed to demonstrate tangible benefits in a prospective assessment. In the HBA, this becomes a problem since—as we argue—the only reasonable, expected and tangible outcome of research can ever be knowledge. The potential long-term benefits on the basis of gained knowledge are unforeseeable and impossible to predict. Research is bound to fall short of these proclaimed societal benefits and its credibility will suffer as long as research has to validate itself through short-term societal benefit. We propose to revise the ethical evaluation based on the HBA and we think it necessary to develop an alternative model for project evaluation that focuses on the value of knowledge as a scientific outcome as a necessary but not sufficient condition for societal benefit.

## 1. Introduction

Concerns about animal research are as old as experimentation on animals itself. When it comes to the use of animals in research, most people seem to share a strong ethical intuition that harm inflicted on animals needs to be justified. This general ethical concern for animals is also reflected in Directive 2010/63/EU [1] “on the protection of animals used for scientific purposes”, which had to be implemented in the legislation of all European member states by 2013. The Directive states that “animals have an intrinsic value which must be respected. Therefore, animals should always be treated as sentient creatures and their use in procedures should be restricted to areas, which may ultimately benefit human or animal health, or the environment.” ([1], recital 12).

In EU member states, animal protection and academic freedom have become two competing, legally guaranteed principles. The HBA is evidence of the fact that academic freedom can be limited because of legal responsibilities towards animals. Every research proposal—“project” in the Directive’s language—entailing procedures on living non-human vertebrates and cephalopods must be approved by competent national authorities through a project evaluation, one mission of which is “to assess whether the harm to the animals in terms of suffering, pain and distress is justified by the expected outcome taking into account ethical consideration and may ultimately benefit human beings, animals or the environment” ([1], article 38d).

The Directive also reflects the wish for a harmonized, consistent, impartial and transparent ethical evaluation tool that gauges both the legal as well as the ethical validity of procedures carried out on animals. Despite the best intentions behind the HBA, we argue that there are several issues with its specification that still require reflection and clarification [2]. Much has been written about the concept of harm and harm assessment [1,3,4,5,6,7,8], but we seem to lack the same level of conceptual and practical clarity as to the understanding of benefit. Specifically, it is unclear what the relation between outcome and benefit is or should be. This is crucial, as the entire idea of the HBA depends on these concepts. What “outcome” and “benefit” are exactly and how they should be “weighed” is in need of clarification to prevent the HBA from becoming no more than a laudable, albeit vague idea that fails due to its lack of applicability.

In the following, we argue that the current idea of a prospective benefit assessment portrays a distorted understanding of the scientific process. It seems to imply a direct causality between project and societal benefit, thereby misconceptualizing the research process and it might incentivize scientists to simplify and overstate the possibilities of obtaining societal benefits. In light of that analysis, we then question what the role of ethical review in the project evaluation process can plausibly be. Should ethical review be reduced to a semi-mathematical exercise that generates answers regarding the justifiability of animal testing in a quasi-algorithmic procedure? Or should it provide guidelines as opposed to specific instructions on how to evaluate the benefit of research involving animals?

This study seeks to analyze the underlying premises of the HBA as outlined in the Directive [1] and their consequences for the evaluation process. To this end, we introduce a heuristic to analyze the relations between “outcome”, “benefit” and “prospective benefit assessment”. We then apply the heuristic to all seven purposes for animal research, which the directive classifies as legitimate: basic research, translational or applied research, product safety, education and training, protection of the environment, preservation of species and forensic inquiries. We methodically analyze and conceptualize the HBA’s semantics as pertains to the benefit dimension set forth in the Directive.

## 2. A Brief Heuristic on “Justified by the Expected Outcome (…) and May Ultimately Benefit”

We argue that the key to understanding the relation between outcome and benefit of an experiment in the HBA lies in one’s reading of the language “is justified by the expected outcome (…) *and may ultimately benefit*”. Upon closer examination of these inconspicuous words, certain implicit premises become apparent.

### 2.1. The Heuristic of “And”

The reader of the Directive’s passage on the HBA will immediately stumble on the question of whether outcome *and* benefit refer to different justifying dimensions. The answer to this question depends on whether “and” directly links outcome and benefit in the passage “is justified by the expected outcome *(…) and may ultimately benefit.*” Following the interpretation that “and” is understood as a direct link between outcome and benefit, a legitimizing outcome would have to be argued to have an expected impact on benefits for humans, animals and the environment. In this reading, either the outcome itself would equate to benefit or the potential consequences of the outcome would have to allow for benefits of the specified nature. Either way, the justifying factor in the HBA would be an outcome that is qualified by its potential to benefit humans, animals and the environment. If “and” does not directly link outcome and benefit, it would suggest that the legitimization of a procedure through its outcome is independent of the outcome’s benefit to humans, animals or the environment. However, in this case “and may ultimately benefit human beings, animals or the environment” would become obsolete and meaningless. Consequently, the only possible and meaningful understanding of “and” is a strong link between outcome and benefit.

### 2.2. The Heuristic of “May Ultimately”

As demonstrated above, outcome and benefit cannot be understood independently of each other in the Directive. We now aim at analyzing the nature of their relation. First, “may” in “may ultimately benefit” indicates that the outcome ought to have the *potential* to be of benefit. Second, the word “may”, especially in conjunction with “ultimately” indicates some time horizon for achieving benefit. Yet, what would count as timely or late remains entirely unclear. Cynics might even argue that it is logically impossible to preclude that any given experiment can “ultimately” (in the far unforeseeable future) yield some kind of benefit for humans, animals or the environment; nobody, one could argue, can say with certitude that a particular experiment will never—not even in 500 years—contribute to some sort of benefit. This would imply that projects eo ipso fulfil the criterion that they *may ultimately benefit*. Although it is clear that nobody can exclude the possibility that a project “may” benefit someone someday, the question remains how substantial the expected benefit is to be, and we are left wondering how such a thing is to be assessed.

The legitimizing power of a procedure’s outcome has been qualified by its expected benefit, and that benefit has been qualified as pertaining to humans, animals or the environment. According to recent publications, catalogues of criteria and guiding documents, the justifying power of benefit is dependent on a) the time frame within which the benefit shall be reached, and b) the probability of the projected benefit’s realization [8,9,10,11,12]. For instance, the questions *“What” is the benefit, “who” will benefit, “how” will they benefit* and importantly *“when” will they benefit?* are highlighted in an EU guiding document [13] and referred to in a recent publication by the “American Association for Laboratory Animal Science” and “European Federation for Laboratory Animal Science Associations” working group (AALAS-FELASA) [9] as important parameters in assessing potential benefits. Prior to these recent publications, published work in the field had been arguing along these lines for years. In 1992 for instance, Porter [5] qualified benefit by its importance for humans and the likelihood of its achievement. Scharman and Teutsch [10] propose a checklist model that defines expected benefit as the improvement and/or development of diagnostics and/or therapies, and factors in the likelihood of its realization as well. Hirt, Maisack and Moritz [12] restrict “benefit” to “benefit to humans with regard to what kind and what extent of benefit and the likelihood and time frame of realization” (authors’ translation). The “expert working group on project evaluation and retrospective assessment” suggests a modified Bateson’s cube [13], an analysis model that dates from a 1986 publication [14] and has served as an ethical evaluation tool for animal research ever since. The Bateson’s cube is a 3-dimensional grid that evaluates the degree of benefit, the amount of harm to the animals and the likelihood of benefit. Not only is the degree of benefit decisive, but also the likelihood of achieving it. Moreover, Bout et al. [11] have developed another matrix based on the Bateson cube, which is used for the HBA in the Netherlands. In their model, the likelihood of the benefit (scientific and/or practical) is more than just a contributing factor in the weighing process; it is a stand-alone criterion. If the likelihood of success is too low, the proposal has to be rejected, regardless of its strengths. Again, preference is given to projects that yield foreseeable benefit. Stafleu et al. [15] proposed a numerical algorithmic model with a detailed set of formulas for calculating harm and benefits. While the authors do acknowledge both the difficulty of prospective benefit assessment and the legitimate value of knowledge as an experimental outcome, only human health interests are capable of getting the maximum score in their evaluation tool. Interestingly, economic interests are accorded the same legitimizing power as the generation of knowledge in the Stafleu et al. model.

In this brief overview of relevant published work on the HBA, a pattern emerges in which the understanding of “benefits” is “foreseeable, expected, societal benefit.” The generation of direct, societal benefit factors more heavily in the HBA and is prioritized in literature on the evaluation process, at the expense of procedures whose intended and foreseeable outcome is of no foreseeable, tangible and immediate benefit to society. In that scheme, generating knowledge (the outcome of basic research) is typically regarded as a lesser benefit and weighs less on the scales of the HBA.

From this, it follows that knowledge per se as the *outcome* of a project is only viewed as a necessary but not sufficient condition to the justification of harm done to the animals. In this dominant understanding of the terms, only an *outcome* which is qualified by its potential to generate societal *benefit*, as direct *benefit* to humans, animals or the environment can succeed to legitimize harm and thus fulfil the legal requirements. Therefore, knowledge, as experimental outcome, is often considered of little justifying power. It follows that, e.g., severe harm cannot be justified solely on the grounds of expected gains in knowledge, as would be the objective of basic research [16,17]. As substantiated above, the prevailing consensus on the HBA evidently locates justifying power not in an expected outcome, but in expected societal and tangible benefit. Here, it is important to note that it is not only important that “benefit” may be generated but that “benefit” should, to some degree, be expected.

Our contention is that the wording “and may ultimately” with regard to “outcome” and “benefit” in the context of the HBA is interpreted in such a way that scientific benefit (knowledge) as an experimental outcome has no or little intrinsic value and legitimizes harm in procedures *only* through its potential to generate societal benefit (i.e., benefit to humans, animals or the environment beyond knowledge). Furthermore, the HBA prioritizes timely and likely (i.e., time frame and probability of success), direct, societal benefit over less tangible benefit, particularly knowledge. We would like to make it clear that we do not claim that knowledge is not a legitimate outcome of animal research according to the Directive. Nor do we claim, that an intention to generate societal benefit (e.g., health or environmental benefit) is a “sine qua non” requirement for project approval. Rather we want to stress that the HBA prioritizes and gives much more “weight” to societal benefits in project evaluation compared to knowledge and prioritizes potential societal benefits that seem more tangible and more likely. The main justifying power of a research project’s outcome is strongly linked to expected societal benefits and thus, gaining knowledge is on the bottom and, e.g., human health on top of the benefit hierarchy in the HBA. In the following, we will argue that this hierarchy is questionable with regards to prospective benefit assessment, due to logic, methodological and practical flaws. We argue that societal benefits of animal research should be assessed retrospectively, but consider it problematic to assess them prospectively in a HBA.

## 3. The Heuristic Applied to All Legal Purposes Listed in the Directive 

We have established that in an HBA the justifying power of a project depends on whether, or to what extent, it fulfils the following criteria:

1. proof that the project *may ultimately benefit* humans, animals or the environment;
2. prospective qualification of the outcome with regards to its potential and expected benefits;
3. prediction of the likelihood and time frame of achieving the benefit.

To ascertain whether the seven purposes for animal research which the directive classifies as legitimate meet the above requirements, we now apply the heuristic to all seven legal purposes specified by the Directive, namely, basic research, translational or applied research, product safety, education and training, protection of the environment, preservation of species and forensic inquiries ([1], article 5). 

### 3.1. Basic Research

Basic research is defined as experimental or theoretical research whose primary intent is to add to or improve knowledge without aiming at any particular foreseeable practical application [18]. The justifying power of basic research depends on the value of knowledge as such, i.e., knowledge independent of application.

#### 3.1.1. Proof That It May Ultimately Benefit Humans, Animals or the Environment

In 1970, Comroe and Dripps [19] analyzed the impact of basic research on the development of the 10 hitherto most influential medical therapies for pulmonary and cardiac diseases. They were able to determine that 2500 publications had contributed to the emergence of one of those therapies, 60% of which was attributable to basic research. A more recent study [20] found that two recently approved drugs, one for cancer (Ipilimumab) and the other for cystic fibrosis (Ivacaftor), were mainly contingent on 433 publications over 46 years and 355 publications over 47 years, respectively (this is only counting the most influential contributions while omitting smaller contributions.). Many of these publications are again attributable to basic research and these findings paved the way for important innovations, showing that basic research can meet the criterion of *proof that it* *may ultimately benefit humans, animals or the environment*.

#### 3.1.2. Prospective Qualification of the Outcome with Regards to Its Potential and Expected Benefits

Because basic research seeks to add to the knowledge base and is not conducted for the sake of any particular practical application [18], most of the studies mentioned above would have been hard-pressed to identify what societal benefit they might yield. If a practical application were foreseen, the study would cease to be basic research. Basic research generates knowledge that is a necessary but not sufficient precondition for the generation of the societal benefit. Basic research is, by nature, afflicted with an immanent uncertainty about what conclusions it will draw and consequently with uncertainty as to what benefit can potentially arise from its findings. That makes predicting societal benefits rather implausible, so basic research fails to meet this requirement.

#### 3.1.3. Prediction of the Likelihood and Timespan of Achieving Benefit

As mentioned, the generation of benefit from research is a non-linear process. The generation of societal benefits is most often neither intended nor foreseeable.

Furthermore, a prospective assessment of benefits only becomes plausible if the outcome of the experiment is known beforehand (e.g., one knows whether a hypothesis will be verified and not falsified). This, however, rests on a logical mistake that neglects the inherent uncertainty in research: If it was already known whether the hypothesis will be verified or falsified, there would be no additional knowledge gained from the experiment. Further, it would be illegal to carry out such research, since it would not result in additional knowledge.

Being unable to foresee benefits, basic research cannot possibly fulfil this criterion, either.

In summary, the outcome of basic research is knowledge as such, which does fulfil the criterion of *proof that it may ultimately benefit humans, animals or the environment.* It fails, however, to meet the requirements for *prospective qualification of the outcome with regards to its potential benefits* and *prediction of the likelihood and time frame of achieving benefit.* If judgement is to be based upon the criteria implicit in the Directive, the expected results of basic research can have little justifying power in prospective assessment. 

### 3.2. Translational or Applied Research

The United States National Institute of health (NIH) [21] defines translational research as “…the process of applying discoveries generated during research in the laboratory, and in preclinical studies, to the development of trials and studies in humans...“. Applied research in the medical field, for instance, seeks to translate aggregate existing findings into medical innovations and advances (“bench to bedside”) [22]. This category of research is more goal-oriented than basic research, which means its potential benefits might also appear more tangible at first glance. Knowledge as such is not only sought, it is sought in light of a hypothesis about a particular application (knowledge as a means to an end). The Directive allows for the use of animals in applied and translational research pursuing any of the following goals: “(i) the avoidance, prevention, diagnosis or treatment of disease, ill-health or other abnormality or their effects in human beings, animals or plants; (ii) the assessment, detection, regulation or modification of physiological conditions in human beings, animals or plants; or (iii) the welfare of animals and the improvement of the production conditions for animals reared for agricultural purposes” ([1], article 5b).

#### 3.2.1. Proof That It May Ultimately Benefit Humans, Animals or the Environment

As shown under point (a), the published findings of basic and applied research have paved the way for great achievements in medicine [19,20]. Applied and translational research effectively bridge the gap between basic research and clinical application (“bench to bedside”) and constitute another important step towards societal benefit. Thus, applied and translational research can fulfil this criterion.

#### 3.2.2. Prospective Qualification of the Outcome with Regards to Its Potential and Expected Benefits

Since applied and translational research are conducted in light of more practical hypotheses and their potential benefits appear more tangible than those of basic research at first glance, it is possible to prospectively qualify the outcome with regards to the nature of its potential benefits, e.g., new cancer treatment. However, the fact that a benefit can potentially be obtained says nothing about whether that benefit can actually be expected and, as we have shown in the heuristic, having the potential to create benefit does not suffice; the decisive factor is that benefits be expected.

The knowledge gained through any experiment is a necessary but not sufficient condition for the generation of societal benefit, and applied and translational research are afflicted with the very same uncertainty as basic research. Yet, a prospective assessment of the expected benefits created by hypothesis-driven research could only be made under the assumption that one already knows whether a hypothesis will be verified/falsified, i.e., what knowledge will be gained. Without knowing the outcome, potential benefit cannot be gauged. However, if the outcome were known, the project would be neither necessary nor legal, since it would not result in additional knowledge. It is inherently uncertain what, if any, gains in knowledge will be made, which means it is uncertain whether potential benefits might be realized. This demonstrates that applied and translational research do not meet the criterion of *prospective qualification of the outcome with regards to its potential benefits.*

#### 3.2.3. Prediction of the Likelihood and Time Frame of Achieving Benefit

Again, the same uncertainties apply here as in basic research. It is also worth noting that non-scientific factors outside the responsibility and competence of researchers complicate the issue of prospective (societal) benefit assessment even further. Consider Susan, an imaginary scientist whose new cancer drug has just been approved for use. She is now confronted with several problems. No pharmaceutical company will produce her drug without financial incentive. Intuitively, one might argue that a definite medical need will automatically yield a sufficient economic incentive. Furthermore, while that probably does hold true more often than not, some approved drugs will never make it to market or are discontinued due to limited market potential [23,24]. Among pharmaceutical flops, we find Exubera, an insulin inhalant which, although effective, was never able to benefit humans. It failed financially due to lack of patient compliance: the inhalator was simply too big [24]. In addition, Orphan diseases present cases in which a definite medical need will not necessarily translate into sufficient market potential (depending on the country, an “orphan disease” is defined by an incidence rate of 1 to 8 in 10,000 people [25]).

The approval of new drugs constitutes a certification of their efficacy and safety. After that, medical professionals and patients still need to be convinced that they should administer or use them, thereby altering their treatment regimen. Decisions of this nature are not always based solely upon medical reasoning; other factors (of particular note: lobbying [26,27]) also come into play in the medical profession. Furthermore, as shown with Exubera, a drug’s efficacy and safety cannot ensure that it will be comfortable, convenient or pleasant for patients. Misuse and/or practical issues are still more reasons that even approved medication will not always end up benefiting humans. Duract, for instance, was a nonsteroidal anti-inflammatory drug that had to be recalled because it caused kidney failure in patients who took it longer than indicated [28].

All of these non-scientific factors clearly impact the likelihood that expected benefits will ultimately be obtained. Even though they can make or break the benefit dimension, these factors are certainly not in researchers’ hands. Altogether, they make predicting the likelihood that, or establishing a temporal horizon by which point benefits stemming from scientific research will be seen, unrealistic. Thus, the criterion of *prediction of the likelihood and time frame of achieving benefit* is not fulfilled*.*

In summary, also the outcome of applied and translational research is knowledge as a means to an end (i.e., cancer treatment), which fulfils the criterion of *proof* *that it may ultimately benefit humans, animals or the environment.* It also meets the requirement for *prospective qualification of the outcome with regards to its potential benefits (e.g., cancer drug).* However, it fails with regards to whether these benefits can be expected. It also fails to fulfil the criterion of *prediction of the likelihood and time frame of achieving benefit.* If judgement is to be based upon the criteria implicit in the Directive, the expected results of applied and translational research can have little justifying power in prospective assessment.

### 3.3. Intermission: Notes on the Distinction between Basic and Applied/Translational Research

At this juncture, it seems fitting to note that we have, in keeping with the Directive’s phrasing, treated basic research and applied/translational research separately. Both politics and the general public maintain a sharp distinction between basic and translational/applied research, and there is a strong bias towards the latter in terms of perceived significance [29]. To a large extent, this probably stems from a presumption that the more concrete benefit-oriented ultimate goals of applied and translational research do in fact generate more societal benefit than do the pursuits of basic research. However, the deeper misconception seems to be that these two branches of research are entirely different matters and function independently of one another.

The parallels between our discussions of basic research and applied/translational research point to the groundlessness of any distinction and hierarchization on the basis of societal benefit, as—when it comes to the generation of societal benefit—there is really no clear line between the two. Both branches of research primarily add to or improve knowledge and, as the outcome of research in general, knowledge is a necessary but never sufficient condition for societal benefit. Furthermore, the poor reproducibility and translational value that is observed in most pre-clinical studies [30,31,32,33] shows that, even though potential societal benefits are more easily identified in applied and translational research compared to basic research, does not mean they can be predicted with certainty. Based on this, we question the hierarchy of benefits from knowledge at the bottom to societal benefit on top.

The low reproducibility and translational value are partly attributed to insufficient scientific standards. Therefore, an alternative model for project evaluation should be developed that shifts the focus in project evaluation from societal benefit to maximizing epistemic benefit (i.e., valid knowledge). To this end, we believe the top priority in project evaluation should be on methodological soundness and scientific standards to reduce bias and gain reliable results (e.g., by blinding, allocation concealment, randomization, adequate sample sizes, appropriate statistics) and applying the 3R’s: “replacement”, “reduction” and in particular “refinement” [34].

### 3.4. Regulatory Tests

Regulatory tests are mandated by legislation. They may be carried out on animals in pursuit of any of the goals listed under point (b) in the development, manufacture or testing of the quality, effectiveness and safety of drugs, foodstuffs and feedstuffs and other substances or products. The justifying power of these tests is based on the qualification of their outcome (knowledge) with regards to its direct application (product safety). The intended outcome is not knowledge as such, but knowledge as a means to an end, i.e., product safety as societal benefit.

#### 3.4.1. Proof That It May Ultimately Benefit Humans, Animals or the Environment

Substances are tested on animal models before being used on target species in an effort to minimize the risk of unwanted effects. The quality, effectiveness and safety of the tested substances can be of great importance for human health, so regulatory tests fulfil the criterion that they *may ultimately benefit humans, animals or the environment.*

#### 3.4.2. Prospective Qualification of the Outcome with Regards to Its Potential and Expected Benefits

The potential outcome of regulatory testing is knowledge directly applicable in the assurance of product safety (societal benefit). To that, we would like to add three remarks. First, even though product safety is a potential ultimate outcome, it remains uncertain whether that outcome can be expected. The points previously made regarding uncertainty in applied and translational research apply here as well. Second, a debate over “luxury” or “unnecessary” products resulted in a European ban on animal testing for cosmetics in 2013 [35], implying that some products are more important than others. If that is the case, there needs to be a comprehensive hierarchical list of products in order of “importance” to ensure consistency. Does the safety of a vaccine constitute a greater benefit than that of a redundant food additive? Or shall we agree to limit testing to “important” products? Then, there needs to be a comprehensive list of all products that fall under that category, and any exceptions or conditions which might affect “importance” need to be established in advance. Conversely, let us imagine that products which have little to add to existing societal benefits, like the 400th sunscreen, score enough justifying power in an HBA to authorize the use of animals. On the grounds of equal treatment of equals, few to no projects could be rejected.

Third, what happens if a legal regulation requires a certain testing regime, but the results of an HBA indicate otherwise?

#### 3.4.3. Prediction of the Likelihood and Time Frame of Achieving Benefit

In regulatory animal testing, the likelihood and time frame of obtaining benefits depend on the translatability of the knowledge gained through animal models to target species. If translatability is high, the time frame for creating benefit will be short. If translatability is low, the time frame for creating benefit becomes difficult to predict. However, translatability has been questioned [36,37] and in strictly methodological terms, as long as animals are being used as model organisms, there will always be uncertainty about the translatability of knowledge gained through animal testing to humans, other animals or the environment. If translatability is uncertain, predicting the likelihood and time frame of benefit creation becomes equally uncertain. Thus, regulatory tests do not fulfil this criterion. We do, however, acknowledge that this mainly holds true for substances that have not been tested before. Substances that have been tested before and have proven to be safe, have been marketed and consumed do not suffer from the same uncertainty regarding translatability (e.g., vaccines that are approved for marketing still undergo batch testing). In this case, the proximity of the animal test to product safety might allow for a plausible prospective benefit assessment.

In summary, regulatory tests do fulfil the criterion that they may ultimately benefit humans, animals and the environment. It is also possible to prospectively qualify the outcome with regards to the nature of its potential benefits, i.e., product safety. The fact that a benefit can potentially be obtained does not bring certainty about when or whether said benefit can be expected, and the creation of benefit in product testing ultimately hinges on the translatability from model to target species, a factor that can vary depending on whether an untested substance or a substance is tested that has already been tested and found safe (e.g., vaccines batch testing). Thus, depending on the substance tested, regulatory tests can meet the requirement of prediction of likelihood and time frame of benefit creation.

### 3.5. Education: Higher Education, or Training for the Acquisition, Maintenance or Improvement of Vocational Skills

The intended and foreseeable outcome of training is knowledge as a means in terms of a particular skill.

#### 3.5.1. Proof That It May Ultimately Benefit Humans, Animals or the Environment

Scientists using animals in their research have greatly benefited humans, animals and the environment many times [19,20,38,39,40]. Medical innovations, for instance, depend on highly skilled and trained scientists. Thus, education and training as a necessary condition for the future use of animals in scientific research fulfils the criterion of *proof that it may ultimately benefit humans, animals or the environment.*

#### 3.5.2. Prospective Qualification of the Outcome with Regards to Its Potential and Expected Benefits

Conducting procedures on animals in the context of training to improve skills and techniques is a necessary condition to ensure, for instance, that researchers and veterinarians are able to perform surgeries, to guarantee maximum welfare for animals and compliance with the 3Rs. The skill or knowledge gained is qualified by its potential to yield benefit upon being put into practice. However, it is unknown whether a trainee will ever actually use the acquired skill in a procedure that might potentially generate societal benefit. For example, only a fraction of veterinary students trained on large animals (e.g., horses, cattle) will ever end up working in that field research or in a large animal clinic. Even if they do, the potential societal benefits of potential future procedures remain unknown. Nobody can know whether a trainee will use her skills to transplant lungs in pigs or to conjoin the circulatory systems of two mice, etc. Although possible criteria for benefit in this context can be identified, uncertainties remain. Is it the number of trained people or the number of instances in which acquired skills are put into practice? The criterion of *prospective qualification of the outcome with regards to its potential benefits* is fulfilled but—on the basis of the reasons given—it can be questioned whether a benefit besides knowledge can be expected with sufficient probability.

#### 3.5.3. Prediction of the Likelihood and Time Frame of Achieving Benefit

As no potential benefits of potential future procedures can be surmised, it is difficult to assess the likelihood and time frame of benefit generation, so the criterion of *prediction of the likelihood and time frame of achieving benefit* seems difficult to fulfil.

In summary, the outcome of training and education on live animals is knowledge also in the form of practical skills. This fulfils the criterion that these endeavors may ultimately benefit humans, animals or the environment. However, it remains implausible in most cases to predict either the nature of potential benefit besides knowledge or when and how likely it is to be realized. Thus, they fail the criterion of prediction of the nature of benefit, the likelihood and the time horizon of realization of benefit.

### 3.6. Environmental Protection, Preservation of Species and Forensic Inquiries

Protection of the natural environment in the interests of the health or welfare of human beings or animals, research aimed at the preservation of species and forensic inquiries all seek to guarantee and/or improve the quality of human and animal life. The outcome is knowledge as a means of guaranteeing and/or improving the quality of human and animal life.

#### 3.6.1. Proof That It May Ultimately Benefit Humans, Animals or the Environment

The knowledge gained from these kinds of inquiry and its value as a necessary condition for guaranteeing and/or improving the health and quality of life of humans and animals mean that these studies fulfil the criterion that they may ultimately benefit humans, animals or the environment.

#### 3.6.2. Prospective Qualification of the Outcome with Regards to Its Potential and Expected Benefits

Potential benefits resulting from all three of these branches of research clearly revolve around improving or maintaining the quality of human and animal life. In the specific case of forensic inquiries, the safety and stability of human society is promoted by solving crimes; potential benefits include proving the innocence of an erroneously convicted person or proving the guilt of a murderer. In the preservation of a species, it is the benefit for humans, animals or the environment that arises from preserving this species. So, all three of these fields fulfil the criterion of *prospective qualification of the outcome with regards to its potential benefits.* However, it remains impossible to predict whether these benefits can be expected.

In the preservation of species, it would be particularly interesting to know whether the degree of perceived benefit depends on the importance of the species—is the benefit greater when a keystone species [41] is saved? Or an endangered species? Or a charisma species [41]?

#### 3.6.3. Prediction of the Likelihood and Timespan of Achieving Benefit

Although the goals pursued in these fields are very practical in nature, the same problems continue to apply, as they apply to research in general. Every inquiry is afflicted with an inherent uncertainty which makes the likelihood of achievement and a time horizon by which point benefit will be seen quite unpredictable. 

In summary, environmental protection, the preservation of species and forensic inquires fulfil the criteria that they *may ultimately benefit* humans, animals or the environment and to *prospectively qualify their outcome with regards to the nature of its potential benefits*. However, they cannot fulfil the criterion of *prediction of the likelihood and time frame of achieving benefit.*

Based on the application of the heuristic to all seven legal purposes of animal use in research listed in the Directive, we conclude that it is possible to identify potential societal benefits for the legal purposes except for basic research which does not have a primarily application-directed goal. However, we question whether societal benefits can be expected and whether they can be plausibly assessed prospectively, especially with regards to likelihood and timeframe of their realization. We argued that the only reasonably expected outcome in animal research for any of the seven legal purposes is knowledge and we therefore object the hierarchy of proposals based on prospective benefit assessments. Regulatory tests of known substances as well as some forms of education might represent exceptions where the proximity of the experiment to application can potentially be close enough to make prospective benefit assessment plausible. However, even in these exceptional cases, there remains considerable uncertainty regarding nature, likelihood and timeframe of benefit generation. 

## 4. Conclusions

### Critique of the Focus on Short-Term Benefit

We conclude that the current conception and implementation of the HBA equate “outcome” with “potential societal benefit”. Projects are qualified by their direct and foreseeable benefits to humans, animals or the environment. The more tangible a project’s expected benefit seems, and the more likely and foreseeable (the sooner, the better) the attainment of that benefit appears to be, the greater the legitimizing power a proposal can gain in an HBA. Yet, the outcome of projects in the areas that constitute legitimate uses of animals in scientific research as stipulated by the Directive rarely meets these criteria. What are the underlying reasons for this paradox? We believe it is mainly the product of a misunderstanding of the scientific process and what can reasonably be expected from good scientific practice. The notion that one single project, or a few consecutive projects combined create foreseeable, societal benefit bespeaks an oversimplification of the complex nature of research. It suggests an illusory, direct and virtually immediate causality between research projects and benefit. We are not arguing that there is no causality whatsoever between research and benefit; science (biomedical) has repeatedly benefited humans, animals and the environment greatly [19,20,38,39,40]. Our point is that the nature of the relationship between research and societal benefit is neither linear nor immediate. The fact that many benefits have only been possible because of research that used animals in no way implies that all such research will bring about societal benefits immediately. As we have argued, the relationship between a project’s outcome and any benefit derived therefrom is less controllable and less predictable than implicitly suggested in the literature we reviewed. Made explicit, the prevailing conception of the HBA applies criteria which cannot be fulfilled due to logical, methodological and non-scientific factors.

The strong bias towards expected societal benefits we identified has already been criticized by others. Mike Lauer, Deputy Director of extramural research at the National Institute of Health (NIH), stresses the importance of basic research as a cornerstone of a diverse science portfolio that actively pursues a thoughtful combination of short-term and long-term goals [42]. Dieter M. Imboden, former president of the Swiss National Science Foundation, protested that the focus on benefit in an ethical HBA represents a paradigmatic shift in legislation, which occurred in the absence of debate on the value of knowledge. He asserts that this is regrettable and fatal ([29], authors’ translation). The shift that Imboden observes was foreshadowed by the cases of neuroscientists Kevan Martin and Andreas Kreiter. In 2006, Martin had to stop his work on macaques in Zurich. The reasoning of the verdict referred to the violation of the animals’ dignity and the lack of foreseeable, tangible short-term societal benefit [16]. Shortly thereafter, in 2007, the local government of Bremen decided not to renew Kreiter’s license to work with macaques because it was “too far from applications” [16,43,44].

We contend that prospective project assessment on the basis of expected benefits is a misguided idea, not on the grounds that expecting benefits is wrong, but because their prospective assessment is highly uncertain, and prediction of benefits is well-crafted speculation at best. That is exactly what the HBA in its present form demands from applicants. In this respect, applicants are at risk of promising too much [45]. Logical, methodological and non-scientific factors can seriously undermine any prospective benefit assessment and contribute altogether to a high degree of uncertainty as to how a particular project may ultimately benefit humans, animals and the environment. These factors radically question the plausibility of the HBA as it currently stands.

We propose to replace the HBA with a “harm-knowledge-analysis” (HKA) for prospective project evaluation and an analysis of the societal benefits in a retrospective evaluation in the form of systematic reviews. In the harm-knowledge-analysis, the inflicted harm on animals would be weighed against and justified by the expected knowledge gain. The importance of the expected knowledge gain would be qualified by its impact on a given research field or research objective (i.e., important human interests). On this account of a possible HKA, the main task of competent authorities shifts from benefit assessment to expected knowledge assessment and the question of whether scientific standards are met to ensure epistemic gain (i.e., knowledge). Thus, the ethical justification on the project level would require two things: First, that a project is carried out in pursuit of significant human interests (identified on the political level). Second the project design has to meet relevant scientific standards. Whether the project’s expected outcome is of high or low justificatory power will be identified through the epistemic gain defined as the expected knowledge contribution to a particular research area. Whether a particular research area is of sufficient importance should be decided on the political level. Here, animal research can be debated and justified in light of benefits assessed retrospectively through systematic reviews. Such systematic reviews would allow for a realistic view of the societal benefits of animal research. The ways and measures to carry out these reviews and whether these benefits are assessed every 2, 5, 10 or even 20 years will naturally depend on the research area in question.

The challenges of our proposal are numerous. First, criteria for valid societal benefits have to be identified on the political level. Second, it needs to be evaluated whether these criteria for valid societal benefits are met in animal research over time. Third, should a research area fail to produce the benefits, the question will arise whether this research area justifies sacrificing animals for human interests. This brings the challenge of how to organize such a public debate and move the ethical justification of animal research from the project level to the political level.

To conclude, we agree that the use of animals in scientific research ultimately has to be justified by the benefits it generates for humans, animals and the environment. However, we caution against legally mandated prospective evaluation that prioritizes short-term societal benefit for the reasons expressed above. Societal benefits should be evaluated by systematic reviews retrospectively and cannot be prospectively assessed plausibly in the HBA. Given that prospective benefit assessment in its present understanding is an exercise in futility, an alternative model for project evaluation that aims to avoid the pitfalls of its predecessor should be developed.
